# Diagnostic Value of Lumbar Facet Joint Injection: A Prospective Triple Cross-Over Study

**DOI:** 10.1371/journal.pone.0027991

**Published:** 2011-11-29

**Authors:** Uwe Schütz, Balkan Cakir, Karsten Dreinhöfer, Marcus Richter, Holger Koepp

**Affiliations:** 1 Department of Diagnostic and Interventional Radiology, University of Ulm, Ulm, Germany; 2 Department of Orthopedics, University of Ulm, Ulm, Germany; 3 Institute for muskuloskeletal Rehabilitation, Prevention and Health Service Research at Charité Universitätsmedizin Berlin, CSSB and CMSC, Berlin, Germany; 4 Spine Center, St. Josefs-Hospital, Wiesbaden, Germany; Statens Serum Institute, Denmark

## Abstract

The diagnosis “lumbar facet syndrome” is common and often indicates severe lumbar spine surgery procedures. It is doubtful whether a painful facet joint (FJ) can be identified by a single FJ block. The aim of this study was to clarify the validity of a single and placebo controlled bilateral FJ blocks using local anesthetics. A prospective single blinded triple cross-over study was performed. 60 patients (31 f, 29 m, mean age 53.2 yrs (22–73)) with chronic low back pain (mean pain persistance 31 months, 6 months of conservative treatment without success) admitted to a local orthopaedic department for surgical or conservative therapy of chronic LBP, were included in the study. Effect on pain reduction (10 point rating scale) was measured. The 60 subjects were divided into six groups with three defined sequences of fluoroscopically guided bilateral monosegmental lumbar FJ test injections in “oblique needle” technique: verum-(local anaesthetic-), placebo-(sodium chloride-) and sham-injection. Carry-over and periodic effects were evaluated and a descriptive and statistical analysis regarding the effectiveness, difference and equality of the FJ injections and the different responses was performed. The results show a high rate of non-response, which documents the lack of reliable and valid predictors for a positive response towards FJ blocks. There was a high rate of placebo reactions noted, including subjects who previously or later reacted positively to verum injections. Equivalence was shown among verum vs. placebo and partly vs. sham also. With regard to test validity criteria, a single intraarticular FJ block with local anesthetics is not useful to detect the pain-responsible FJ and therefore is no valid and reliable diagostic tool to specify indication of lumbar spine surgery. Comparative FJ blocks with local anesthetics and placebo-controls have to be interpretated carefully also, because they solely give no proper diagnosis on FJ being main pain generator.

## Introduction

In 1911, Goldthwait [1] suggested lumbar facet joints (FJ) as a source of low back pain (LBP). Ghormley [2] coined the term “facet joint syndrome” (FJS) in 1933 and was the first who described the combination of symptoms caused by lumbar FJ degeneration. In 1954, Hirsch et al. [3] described the possibility of FJ pain provocation by injecting saline solution intraarticularly and thus evoking “memory pain”. Mooney and Robertson [4] reported that experimentally caused pain can be relieved by injecting local anesthetics (LA) into FJ, which was the basis for a diagnostic and therapeutic procedure, the so-called facet joint block (FJB). Today, lumbar FJS is widely used as a clinical diagnosis even though there is is still discussion in the literature, whether it is an actual disease or a symptom [5], [6], [7], [8], [9]. This diagnosis is mostly made by exclusion of other causes of pain.

As the intervertebral discs are compressed in the upright position, the facet joints are subject to compression under (hyper-) extension of the spine. The amount of force was calculated at 16% (3–25%) of the entire compression force of the lumbar spine [10], [11]. The strain of the lumbar FJ is highest in maximal extension. The reduction of disc height also increases the load [12], [13], [14], which leads to degeneration of the FJ [15], [16].

Autonomous nerves in FJ have been proven to explain the role of the FJ in LBP [17]. However, the role of nociceptors is still discussed controversially [18], [19], [20], [21]. A dual innervation of the FJ (posterior branches from the same segment and the adjacent cranial segment) was proven, which explains overlapping zones of referred pain [22], [23], [24], [25], [26]. Main factors of degeneration are age, height of the respective segment, especially in case of the lower lumbar segments, and the FJ angle [27].

Schleifer et al. [28] developed a clinical score for grading the discomfort caused by FJS, which included the parameter finger-floor distance, lumbar spine rotation, Schober's index and the 10 point visual analog scale (VAS). Helbig [8] suggested that LBP decreasing with extension and rotation in combination with degenerative FJ changes in x-ray had a high correlation with FJ pain. Schwarzer et al. [29], however, dismissed the criteria reported by Helbig [8]. Correlation between radiological imaging techniques and clinical findings and the distinguishing between symptomatic and asymptomatic individuals using conventional x-ray, CT-and MRI scan or SPECT) is limited and unreliable [30], [31], [32], [33], [34].

Because of unspecific and inconsistent clinical symptoms (local and pseudoradicular pain) [5] and a low predictive value of diagnostic imaging, interventional tests for detection of degenerative LBP are required [33], [35], [36], [37]. The indication for diagnostic FJB by facet joint injection (FJI) using LA is therefore to numb a FJ and thus to identify it as the origion of pain. Residual pain after FJB is not attributed to the injected joint [34].

In literature [38], [39] the following responder or predictor criteria were mentioned to indicate a positive response to a FJB: advanced age, history of LBP, no leg pain, Valsalva test negative, no muscle spasms, normal gait, and increasing pain following flexion. None of these specific studies were able to produce a significant predictive value or parameter (anamnestic, clinical, functional) [40], [41].

The prevalence of a disease defines the importance of correct test results. Therefore, diagnostic tests in a population with high prevalence of a specific pathology are more important than in populations with a lesser frequency of the same pathology. Some investigators, with limited evidence and no gold standard, suggest that the prevalence of FJ involvement in LBP seems to be 15–40%, with LBP caused solely by the FJ as low as 7% [38], [42], [43].

The FJI as diagnostic tool for FJS is a widely accepted instrument in spine surgery for preoperative diagnostic. The results are often used in decision making for FJ denervation, segmental dynamic or rigid stabilization [44].

The property clinicians expect most from a diagnostic test is a good predictive value, which is a function of the specifity, sensitivity, and validity of the test applied, as well as safety (low rate of complications) and reliability (reproducibility). It is considered impossible to accurately determine specificity and sensitivity in spine related issues [35]. For diagnostic tests such as FJI with the aim of the test being presence or absence of pain, there is no reliable gold standard.

There are numerous reports on diagnostic lumbar FJB [5], [25], [25], [40], [41], [42], [45], [46], [47], [48], [49], [50], [51,52]. However, the aplied techniques vary widely and therefore their comparability is limited. FJI without radiologic control of needle positioning lacks precision and shows a higher risk of complications [53]. Jerosch et al. [52] were able to show in a human cadaver study that the median inaccurateness is 2.3 mm for injection under x-ray control. The highest specificity was reached with MRI or CT guidance [35], however, effort and costs lead to a preference of fluorescent imaging [50]. The risk of FJB is generally low, although there are case reports on infections [45], [54], [55], [56], [57], [58], [59], [60].

### Purpose

Randomized studies have been published in which either placebo or verum was injected [41]. However, due to the complexity of the symptoms, it is preferred that both agents are injected in the same patient. A “cross-over design“ regarding these necessities has not been published to date. The present study examined whether a lumbar FJI is suitable to identify FJ's as pain originating structures or whether optimization of the preclinical testing is necessary to enhance the probability of the prevalence. The problem of placebo effects is also addressed.

## Materials and Methods

### Design

A prospective, clinical, randomized, closed, single-blinded, triple cross-over study with six parallel groups was performed. Verum agent and placebo were applied as intraarticular FJI in all patients. According to study protocol, each patient received three bilateral injections: verum (V: 1.5 ml 1% Mepivacaine), placebo (P: 1.5 ml 0.9% isotonic sodium chloride solution) and sham injection (S: only extraarticular positioning of the needle without volume application, in order to avoid irritation of the joint capsule) after a period between 8 and 12 hours (wash out period). If the patient was still reporting a benefit from the previous injection, the following injection was also performed, however, the current pain level was taken as base for the following injection. For the injections, gauge 22–23 needles with a length of 5–15 cm (3.5–5 in.) were used. In order to avoid additional irritation, local anesthesia of the skin or contrast medium application were avoided.

The order of injections ( = sequence) was randomized according to a protocol created by the local Department of Biometrics and Medical Documentation leading to 6 evenly split sequence-groups (table 1).

**Table 1:** Sequences (groups of possible injection series).

	1^st^ injectionA	2^nd^ injectionB	3^rd^ injectionC
**1. group (n = 10): SPV**	sham	placebo	verum
**2. group (n = 10): SVP**	sham	verum	placebo
**3. group (n = 10): PSV**	placebo	sham	verum
**4. group (n = 10): PVS**	placebo	verum	sham
**5. group (n = 10): VPS**	verum	placebo	sham
**6. group (n = 10): VSP**	verum	sham	placebo

### Patient sample

60 consecutive patients admitted to the local orthopaedic department for surgical or conservative therapy of chronic LBP were included in the study. These patients had undergone adequate conservative outpatient treatment for chronic LBP for at least 6 months without success. The mean age (31 f, 29 m) was 53.2 years (22–73).

Exclusion criteria were age under 18 and over 75 years, intermittent LBP triggered solely by stress, pain during the night or mainly ischialgia with positive Valsalva or Lasègue test. Patients with radicular pain referable to the same segments of FJ degeneration were not accepted. Patients with maximum local pain in the thoracolumbar region, sacroiliac pain or with a history of major surgical procedures of the lumbar spine (like dorsal instrumentation, intervertebral fusion, dorsal spondylodesis, disc prosthesis), osteonecrosis, tumor or severe anatomical deformities, local or systemic infection, tendency towards bleeding, severe osteoporosis, metabolic bone disease, kidney failure, obesity (Broca index >30), pregnancy, allergy to any of the agents applied, or poor compliance were also excluded from the study.

**Figure 1:**
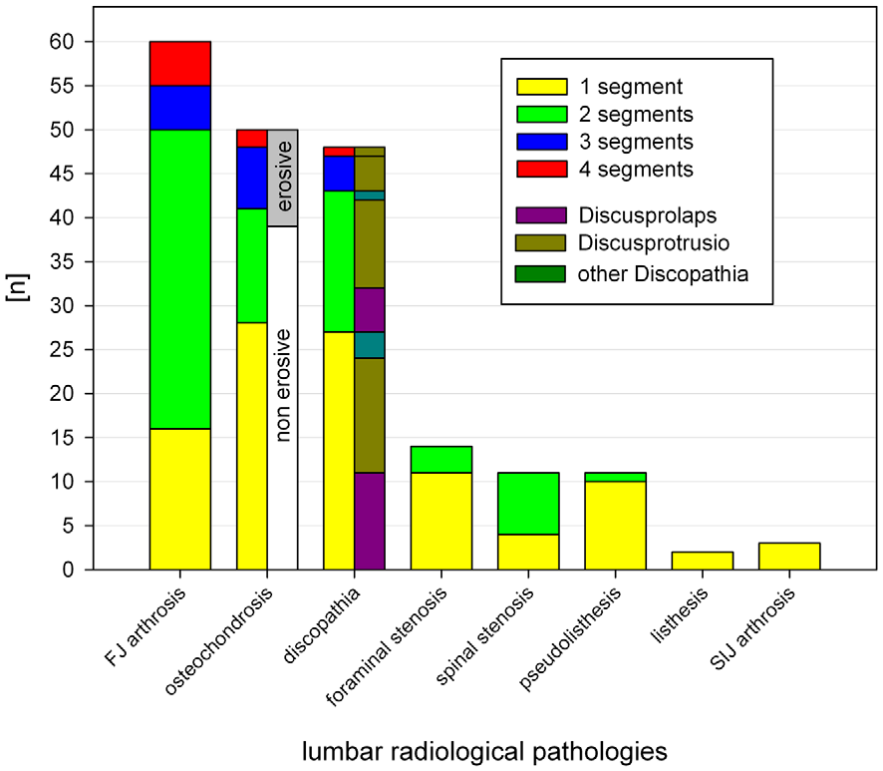
Radiological findings (MRI, CT, X-ray).

Patients fulfilling these criteria had their medical history taken and underwent a physical examination including evaluation of radiological imaging (plain film radiographs of the lumbar spine in 2 views, CT and MRI scans) according to the criteria introduced by Helbig [8], Fairbank [61] and Schleifer et al. [28]. All patients had standard x-rays of the lumbar spine in 2 planes (a.p. and lateral), 91.7% of the patients had MRI of the lumbar spine and 8.3% a CT scan, also. For radiological findings, see figure 1. Radiological entities diagnosed on radiographic images of the LS are multifold and most patients have had more than one. All patients showed degenerative facet joint arthritis in the injected segment, 56.7% in 2 segments and 16.7% in more than 2 segments. Pain pattern showed local lumbar back bain and/or pseudoradicular pain in the buttuck or groin. In 4 patients (6.6%) a selective nucleotomy (minimal invasive surgery) was performed more than 18 months ago. In 3 of these cases, the operated segment was the segment of injection, however, a typical postnucleotomy syndrome with intraspinal scar tissue or instability was not present at time of investigation. On contrast enhanced MR images of LS peridural adhesions or neuronal alteration due to scars in the spinal or neuroforaminal regions were not detectable and they did not suffer from radicular pain in the last 12 months. They only suffered from focal (lumbar, lumbosacral) or pseudoradicular (gluteal) pain.

If a degenerative FJS was present, the patients were informed about the content and course of the study and written informed consent was obtained with approval of the local ethics committee (University of Ulm, Ethikkommission, Helmholtzstr.20, 89081 Ulm, Germany, No.: 10/04-UBB/se.) and in accordance to the Declaration of Helsinki. The patients were admitted to the study according to the randomization plan when the current pain was adequate (VAS>4) and no analgesics were taken 24 hours prior to the first scheduled FJI.

All patients had local lumbar back pain, 44 patients (73.3%) had pseudoradicular pain additionally. Another 11 (16.7%) patients had intermitted radicular pain, which could be referred to spinal segments above the indentified segments of FJ degeneration (all L5/S1 in this subgroup). Pain had been present for an average of 31 months, with a range from 6 months to >10 years. In only 2 cases (3.3%) the level L3/4 was injected, otherwise FJI was done in segments L4/5 (46.7%) or L5/S1 (50%).

### Injection technique

**Figure 2:**
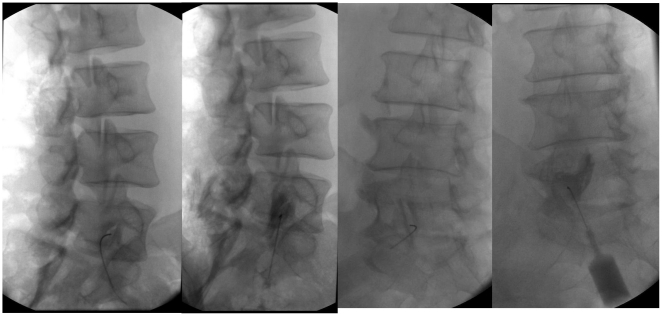
Oblique needle technique of fluoroscopically guided lumbar fact joint injection.

In order to optimize fluoroscopy of lumbar FJ, we used the “oblique needle technique“ (figure 2): the patient was placed in an oblique prone position on the x-ray table, under consideration of the particular lumbar facet joint anatomy [57], [62]. The optimal obliqueness for the upper lumbar FJ is 30°, for the lower FJ approximately 60° [48], [63]. This ensures an orthograde projection of the lateral portion of the FJ. Several authors [44], [48] have identified this needle position to be best in order to avoid iatrogenic cartilage damage.

For the exact diagnosis of pain syndromes relating to FJ, it is considered as a crucial factor, that the injected agent is not be allowed to diffuse into adjacent structures. Therefore, the joint capacity must not be exceeded and the tip of the needle has to be positioned intraarticularly [46]. The volume of the joint capsule has been determined to be 1–2 ml [64], [65]. Excess of this volume will lead to a rupture of the joint capsule and extravasatation [48], [63].

In 60% of cases, the injection sequence was done within 2 days, and 30% within 3 days. In 6 subjects (10%), the testing period included a weekend which led to an extension of the testing period to 4 days. During the study, no patient withdrew from the testing.

### Statistical analysis

In this triple cross-over design, the interesting factor was the effect of the injection. The subjects were blinded toward the applied injection sequence. Target value was the pain level and the change of pain intensity after FJI. The pain level was recorded using a 10-point visual analogue scale (VAS: 0–10) before the injection (time t0) and at definite time t1 (30 min), t2 (60 min), t3 (2–3 h) and t4 (6–8 h) after the injection. If degenerative FJS were present in 2 or 3 segments, the segment which was clinically or radiologically most likely affected was chosen. If a clear identification was not possible, the statistically most likely affected segment L4/5 was tested [34]. The latter occurred in only 1 case, on all other 43 cases with more than 1 segment of FJ arthrosis (figure 1) a segment with more severe degenerative condition could be identified on radiological images.

Evaluation included description of the collective, dropouts and comparison of sequences and descriptive statistical evaluation. To gerneralize the effect of triple cross-over injection test design the data results were evaluated using a linear mixed model. For time t1–t4 a special model was calculated (variance-component-model). Target value was the difference of the score value before the treatment (t0) minus the score value after an injection, separate for t1, t2, t3 and t4. Cause variables were the sequence of injections and pain level before injection. Disturbance variable was the time between injections.

The test injection was considered positive if the difference of the pain scores 2 pts (VAS) in minimum. In case of pain difference below 2 pts or an increase of pain compared to the pain level before FJI the test injection was regarded as negative. In other words; a responder reacts to an injection (V, P or S) with a relevant reduction of pain of at least 2 pts (Δ≥2), a pain relief of less than 2 pts after injection (Δ<2) is defined as non-response.

With regard to the cross-over design, we checked for the presence of a sequence (periodic) or carry-over effect. In this model, the subject was considered incidental and, therefore, the combined structure of the data was taken into consideration. All calculated models were viewed as an explorative data analysis [41].

With regard to the carry-over effect, the tests for difference and equivalence were based on the following hypothesis: the physiological effect to the verum is not influenced by previous injections (H_0_). With regard to the periodic effect the following hypothesis was constructed: the physiological effect to the verum is not influenced by the time of injection (H_0_).

For implementation of the testing on equivalence [41] of different injections, the following hypotheses (H_0_) were constructed: the 3 injections are not equivalent, therefore at least one comparison of effects of two injections would result in a difference more or less than Δ = 2.

In order to enable comparison regarding the equivalence of interventions, the differences of the estimated median values (verum vs. placebo, verum vs. sham, placebo vs. sham) were calculated with the 90% confidence intervals. If all 3 confidence intervals were within the clinical relevant equivalence interval (Δ≤+/−1 equivalence of the 3 different interventions (V,P,S) with a significance of p = 0.05 [41] is proven. If H_0_ is correct, this means that the diagnostic FJI with verum is a sufficiently specific method for differential diagnostic testing of a degenerative lumbar FJS.

The results were descriptively analyzed and evaluated using the following differentiation (table 2): A total non-responder does react to any injection with a pain relief less than 2 pts. (Δ<2). A true verum-responder is given, when reaction to verum is at least 2 pts better than to placebo (ΔV - ΔP≥2) and when reaction to sham injection is not positive (ΔS≤0). A false positive reaction of a verum responder is given, when reaction to verum is less than 2 pts better than to placebo (ΔP - ΔV≥−1) or when reaction is positive to sham injection (ΔS>0). Placebo effect or sham injection gives better pain relief than verum, when difference is positive compared to verum (ΔP - ΔV≥1, ΔS - ΔV≥1).

**Table 2:** Definitions of responder criterias.

total non responder	true verum responder	“false positive” verum responder	placebo responder better than verum	sham responder better than verum
ΔV<2andΔP<2andΔS<2	ΔP+2≤ΔV≥2andΔS≤0	ΔP+2>ΔV≥2orΔS>0	ΔV−1≤ΔP≥2	ΔV−1≤ΔS≥2

Tests on difference regarding the change of pain level (10 pts-VAS) between the 3 injection forms (V,P,S) at the different times after FJI and between the 3 severity groups of FJS (classification of Helbig [8] and Schleifer [28], table 3) were done, using signed-rank tests for dependent ordinal scaled samples: the Friedmann test for test on difference between all 3 types of injection (V,P,S), the Wilcoxon-Test for paired test on difference (V vs. P, V vs. S, P vs. S).

**Table 3:** Severity of facet joint syndromes.

Scores:	Helbig et al. [8](total: 100 pts.)		No. in sample [n]	Schleifer et al. [28](total 15 pts.)		No. in sample [n]
**Stage 1**	≥60 pts.		37	5–7 pts.		33
**Stage 2**	≥40 pts.		27	9–11 pts.		23
**Stage 3**	<40 pts.		6	12–15 pts.		6
	**subgrouping**	**Pts.**		**subgrouping**	**Pts.**	
	Groin or thigh pain	30	44	FGD (finger ground distance)	1: >20 cm2:10–20 cm3: 0–10 cm	21318
	Paraspinal tenderness	20	15	Schober's index	1: 0–2 cm2: 2–5 cm3: >5 cm	232413
	Pain in extension-rotation	30	36	Rotation LS	1: fixed2: limited3: >20°	143511
	Typical radiographic changes	20	60	Lumbago VAS	1: >52: 3–53: 0–2	45132
	Pain below the knee	−10	11	Pseudoradicular VAS	1: >52: 3–53: 0–2	29114

For all tests the level of significance was set on p = 0.5.

## Results

### Carry over and periodic effect

**Figure 3:**
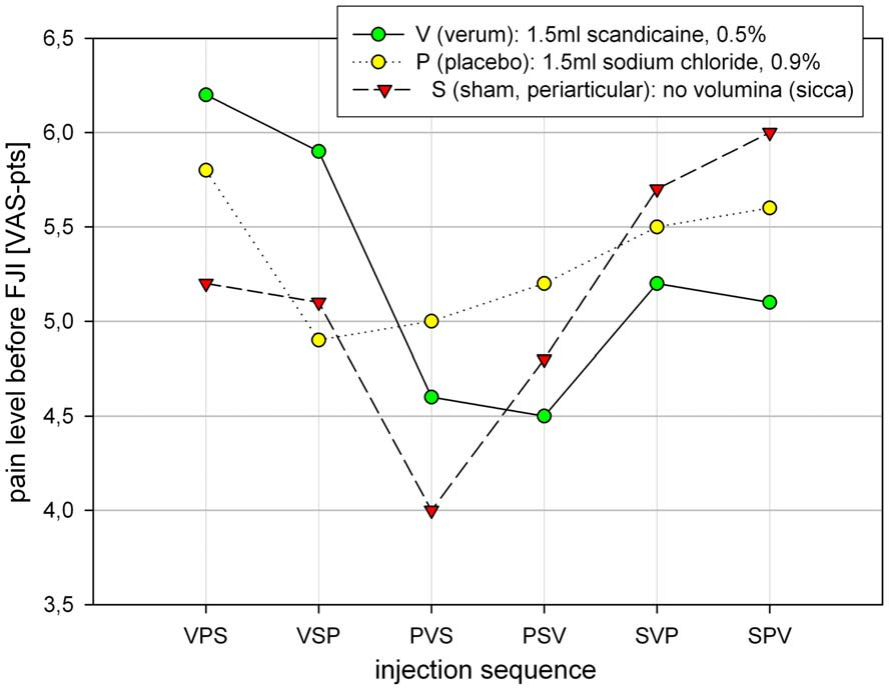
Carry over effect in sequences.

**Figure 4:**
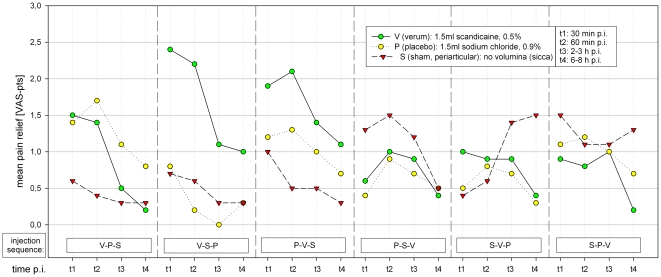
Period effect in sequences.

At no time after injection (t1–t4) a carry over effect could be demonstrated which means, that no injection type had any influence on the subsequent injection (Table 3, Fig. 3) At time t2 there was a significant periodic effect (p<0.042), while at all other times after injection this effect was absent (table 3, figure 4).

### Pain relief

**Figure 5:**
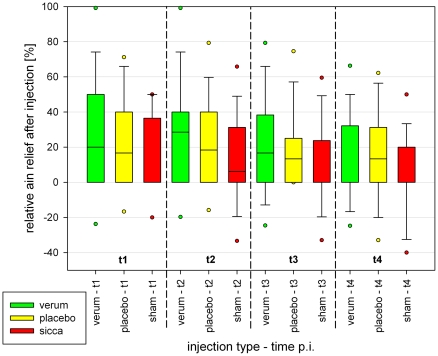
Relative pain relief after FJI.

The distribution of the pain reduction caused by the injections in relation to the pain level before the injection is shown in figure 5. Thirty to 60 minutes after veum-injection the mean pain reduction was 1.4 pts, later below 1 point. With a mean pain relief of 1.2 pts 60 minutes after FJI the effect for placebo-injection was nearly on the same level. During the entire time period up to 8 hours after injection, the mean pain reduction after shame-injection was lower than after verum or placebo; at 30/60 minutes approximately 0.6–0.7 pts. Later, the mean difference between shame and verum- or placebo-injection decreases considerably.

### Responder rates

**Figure 6:**
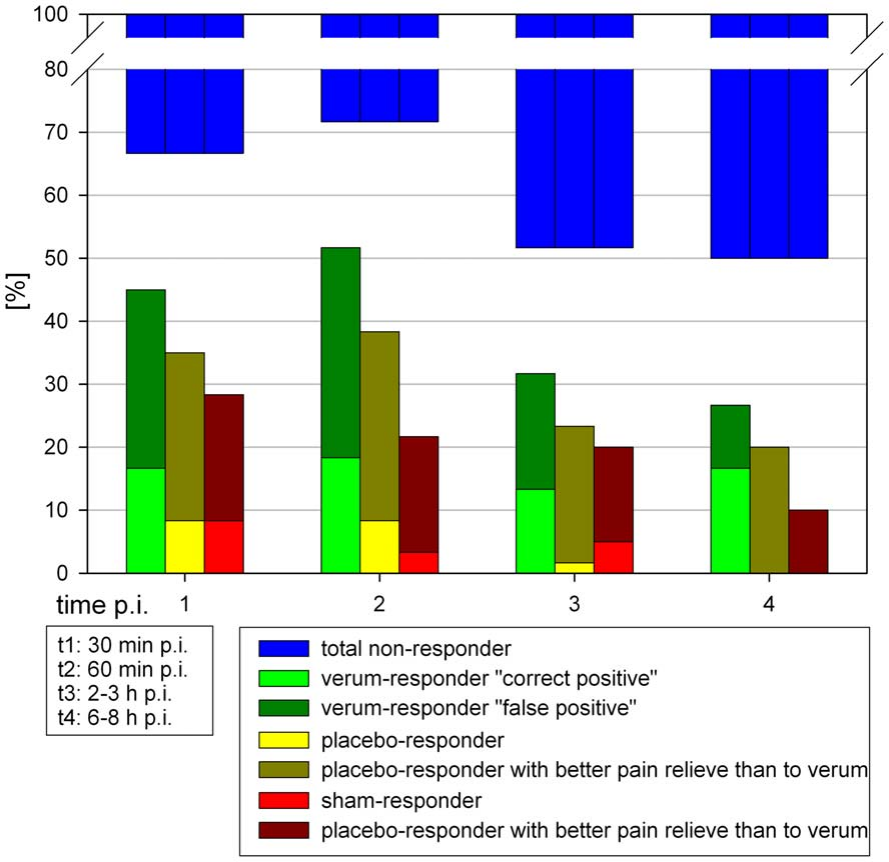
Specific responder rates.

Responder rates regarding verum, placebo und sham-FJI are depicted in Fig. 6: Among the entire sample at 30 and 60 minutes after FJI, 33% respectively 28.3% had a total negative response to any diagnostic FJI (total non-responder). At these times after injection, 67.5% resp. 71% of the responders have been verum-responders, however, in 2 out of 3 cases the placebo or sham values were better (“false positive”). At t1, t2 and t3, approximately 50% of responders were placebo responders and 25–30% were sham responders, indicating that the placebo effect in the majority of the patients (>80%) led to more pain relief than the verum. While at t1 the sham responder rate was 30% (42.5% of all responders), at t2 the rate was 20% (30% of responders). After 2–3 hours the non-responder rate was nearly 50%. Only 2 out of 3 subjacts at this point were verum responders. In cases with equal reaction to placebo- and sham-injection, the pain reduction was almost always equal or better than to verum. False negative responses were not observed which means that there was no increase in pain level following verum injection and pain reduction following placebo- and/or sham-injection.

### Test on equivalence

**Figure 7:**
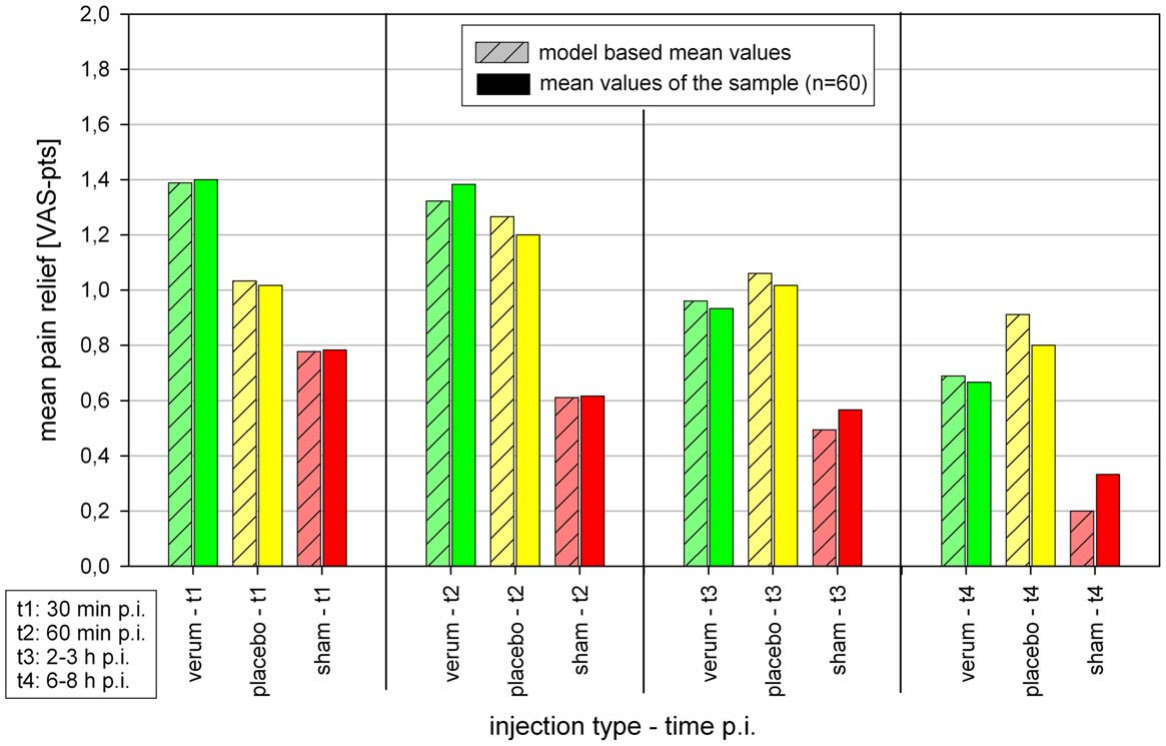
Comparison of model based estimated and measured mean values. Assumption: Mixed linear model and connection structure of data (the patient is regarded as coincidental in the model). Random sample.

**Figure 8:**
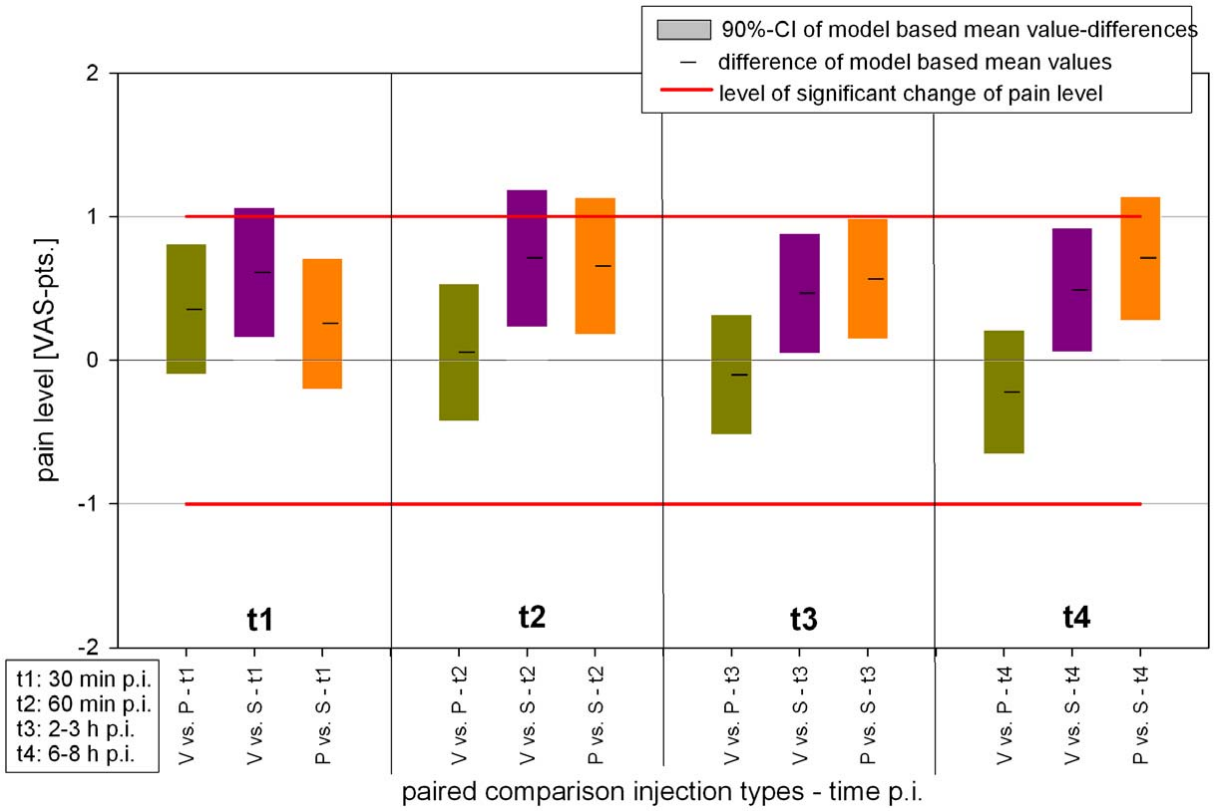
Test on equivalence of injections.

The estimated model based mean values of the target value with regard to the different injection types are depicted in figure 7, showing similar values of measured means of the sample collective. The tests on equivalence showed a significant result for the equivalence of verum vs. placebo FJI at each time after injection (t1–t4), because all 90% confidence intervals of the estimated means of the target value were within the limits for a relevant change of pain level (Δ>1). This is shown in figure 8 by the difference of the estimated means. For sham injection the test on equivanlence vs. verum or placebo was partly significant at time 2 and 4 after FJI. Therefore, the test of difference in pain relief indicates some significant difference for verum or placebo injection vs. sham injection at the same times after FJI (table 3).

### Tests on difference

**Figure 9:**
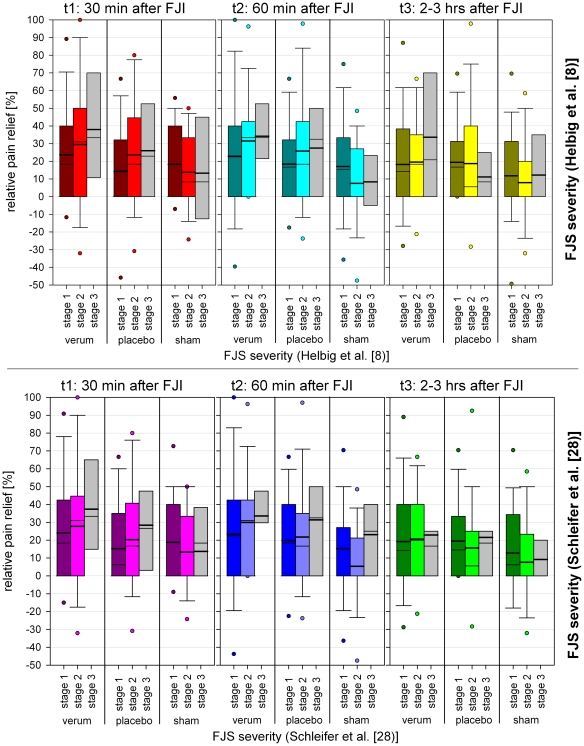
Relative pain relief after FJI in dependence to prior FJS severity.

There is no significant difference of pain reduction between verum- and placebo-injection at any time after FJI (table 4). Between sham-injection and verum- resp. placebo-injection there is significant difference only 60 minutes after FJI (table 4). Figure 9 indicates pain relief between the different stages of FJS severity at time 1,2 and 3 after FJI. As the wide spread indicates, the tests on difference between the severity for stage 1,2 and 3 (classification of Helbig [8] and Schleifer [28]) regarding significance of pain relief are not significant at any time after FJI and for any type of FJI (not for V, not for P and not for S). Tests on difference of patients with stage 3 severity were not possible due the low number in this sample (n = 6, table 3).

**Table 4:** Tests on difference.

effect	t1	t2	t3	t4
**carry-over effect**	**0.788**	**0.535**	0.566	0.138
**period effect**	**0.073**	**0.042** *	0.598	0.813
**pain relief**				
**V vs. P vs. S**	**0.087**	**0.026** *	0.060	0.023*
**V vs. P**	**0.132**	**0.11**	0.108	0.098
**V vs. S**	**0.087**	**0.034** *	0.089	0.065
**P vs. S**	**0.077**	**0.045** *	0.097	0.021*

*p = 0.05.

t1: 30 min after FJI, t2 60 min after FJI, t3: 2–3 h after FJI, t4: 6–8 h after FJI.

### Prior nucleotomy

In 3 of the 4 patients with prior nucleotomy a positive pain relief is seen after verum and placebo at time 1 and 2 after injection, 1 patient showed no response to all injections. In 2 patients pain relief is measured after sham injection at time 1 and 2 also. Due to the small number of 4 patients statistical analysis on difference to patients without prior operative intervention was not possible.

## Discussion

### Equivalence of verum and placebo

Early studies of FJI with steroids and anesthetic agents for diagnosis and therapy were encouraging; short-term pain relief from 59% up to 94% and long-term pain relief from 27% to 65% after a single injection were reported [23], [47], [66]. This led to the conclusion that FJB is simple, safe, and cost-effective, and the technique should be used in the management of LBP. However, many of the early studies were uncontrolled. Studies have shown that even without a placebo effect, one does not have to block the actual painful site of pathology directly to have subjective pain relief [67], [68]. In a prospective, controlled study of FJI, Lilius et al. [69] tested 109 patients with chronic LPB. The patients randomly received one of three types of injections: corticosteroid and local anesthetic into two FJ, the same mixture periarticular of two FJ, or physiologic saline into two FJ. 64% of the patients reported initial relief of pain, and 36% had benefits persisting up to 3 months. The benefit was independent of the mode of treatment given; results in patients injected with saline were as good as results in those injected with local anesthetic and steroids. It was therefore assumed that the mode of injection did not affect the outcome, but the outcome correlated closely with the results of the psychosocial tests.

We were able to confirm these results with a controlled triple cross-over design. At time point 2 the periodic effect and thus the test for difference became significant (table 4). This result, however, does not contradict the significant results of the tests for equivalence between verum and placebo, because the estimated means for therapy, carry-over effect and periodic effect were adjusted [65].

### Lack of clinical predictors

The lack of reliable clinical predictors with regard to FJ pain on the lumbar spine was confirmed by our relatively high rate of non-responders of 33%. The rate for positive tests (true verum responder) was only 16.5% (figure 6). Moran et al., 1986 [51] got the same results with their setting: 54 patients, 143 FJI, prospective, strictly intracapsular, test positivity at pain provocation (0.5–1 ml anionic contrast medium) and relief (<1.5 ml bupivacaine). Unfortunately, due to lack of a control group and only unilateral injections, their results are only interpretable in part. Raymond and Dumas, 1984 [44], used a strictly intracapsular injection technique with 16% positive results, also. Our results for positive NaCl reaction were also confirmed in the literature: in one study 30% of individuals receiving subcutaneous or saline injections rather than lumbar facet joint blocks experienced relief of their facet joint pain [43].

In our sample, classification of clinical and radiological findings in FJS severity scores like suggested by [8] and Schleifer [28] indicated no significant difference in pain relief, not for verum and not for placebo FJI. Like Schwarzer et al. [29], we have to conclude, that these scores are really doubtful regarding validity and therefore, they are not notacceptable for application in diagnostical or therapeutical procedures in clinical practice. North et al., 1996 [66] tested three different nerve blocks by blinded patients in a randomized sequence compared to a control lumbar subcutaneous injection of an identical volume of 3 ml of 0.5% bupivacaine. There were no associations between the results of blocks and clinical findings (history, physical examination, diagnostic imaging) in these patients, chosen for their homogeneous clinical presentation and absence of functional signs. Their results confirmed the hypothesis that false positive results are common and specificity is low. This lack of specificity may, however, be advantageous in therapeutic applications, but this is not proven by cross over studies like our till now.

### Extravasation

The large volumes injected in the early investigations almost certainly resulted in capsular rupture with extravasations of local anesthetic and steroids. In many reports the volume of the injected LA exceeded the capacity of the FJ by far, thus increasing the probability that the effect of FJB was due to other, extraarticular mechanisms [51]. Raymond and Dumas [44], in a study of 25 patients, prevented extravasations by restricting the total volume of fluid injected to 1 ml. Overall relief of pain in their series differed dramatically from that in other series—16% temporary relief and no long-term relief. Moran et al. [51] restricted injection volume; they achieved only a 13% success rate. This extravasate was found in the epidural space rather than in the paravertebral tissue [51], [65], thus leading to epidural and/or segmental nerve root blocks [52]. The positive effects of epidural blocks are well documented [52] and may result in a reduction of pain which is considered a positive effect [24]. A differentiation of the effect as wanted for diagnostic test blocks was not possible.

Lynch and Taylor [70], however, contradict the work by Raymond and Dumas [44] and Moran et al. [51]. They administered two injections of 1 ml of fluid containing corticosteroids in 50 patients. Patients were classified as having both, one, or neither of the injections put into the capsule. The results showed that intraarticular injections were more effective than extraarticular injections for long-term pain relief. There were no control groups.

We performed intraarticular test injections with local anesthetics and contrast medium (total volume 1.5 ml) in 8 patients which were not part of the study group applying the same technique and found extravasations in half of the patients (4) even though the needle was placed correctly (figure 2).

### Prior nucleotomy

LFJ syndrome is a known possible consequence after nucleotomy, due to mechanical pathological load resulting from loss of intervertebral distance [28]. Like the patients without operation at LS in their history, the patients with prior nucleotomy (minimal invasive intervention) show positive pain reduction in 75% of cases also. One of these patients is a total non-responder, like 30–50% of non operated patients are. Compared to prior non-operated subjects, descriptive analysis shows no difference in response reaction on injections, although a statistical analysis on significance was not possible due to small number of this group.

### False positive reactions

According to our results a single intraarticular FJI does not confirm the diagnosis of a FJS. We found a low specificity (high rate of 66% false-positive verum responders) and a low sensitivity (high rate of positive sham reactions in patients with negative verum reaction). Other investigators suggest the reproducibility of the single (uncontrolled) injection is not high, and the specificity may be about 65%. Schwarzer et al. [71] clearly showed in a controlled study that a single diagnostic FJB carries a false-positive rate of 38%. However, the anesthetic response to a single uncontrolled FJB is as high as 50%. The argument has therefore been made that a single uncontrolled facet block will inherently have an unacceptably high false-positive rate and a low positive predictive value [71].

Jackson et al. [38] performed an elaborate study on 390 patients in which they used intraarticular injections of only 1 ml of 0.5% bupivacaine and 2 mg (0.5 ml) of triamcinolone. The investigators evaluated 127 variables and found that more pain relief was associated with older age, history of LBP, normal gait, maximum pain on extension after forward flexion in the standing position, absence of leg pain, muscle spasm, and aggravation of pain when the valsalva maneuver was performed. The authors concluded, however, that the FJ were not commonly the single or primary cause of LBP in most patients. Therefore, it can be assumed that patients with positive test result do not only have pain originating from FJ, but from co-factors which cannot be identified due to the lack of clinical predictors.

### Placebo

Figure 6 shows that 3 and 6–8 hours after FJI a similar amount of patients were true verum responders as 30 and 60 minutes after FJI. Since the local analgesia following mepivacaine injection is reported to content 3–4 hours [72], a positive effect after 5 hours is not only due to verum reaction but also other factors. Thus placebo reactions are to be expected in verum responders from our results as well.

In cases of (sub-) chronic LBP we can assume that a greater rate of patients already suffer from somatic pain disorders, which would explain a certain influence of placebo reactions on verum injections caused by psychosomatic factors. Temporary “diagnostic” nerve blocks may be nonspecific in localizing pathology which generates or maintains an ongoing chronic pain problem [66].

### Sham injections

In literature, periarticular sicca sham injections in lumbar FJ is not discussed or compared to volume injections till now. At time 2 and 4 after FJI the sham injection partly shows relevant difference to verum and/or placebo. Relevant relief of pain level was set as Δ>1, This is very low; with a level of significance of Δ>2, the injection types (V,P,S) would have been equal at any time after FJI. Equality of extraarticular sham injection without volume to the intraarticular volume injection was significant in most but not all cases in our sample. But tendency of lower placebo effects of sham injection compared to placebo injection could be shown. Further inverstigations should confirm the hypothesis, that intraarticular volume application in lumbar FJ influences pain perception resp. placebo effects more than perifocal simple sham therapeutic procedure.

### Gold standard

It is essential to have a gold standard with which to compare the accuracy of a given diagnostic test. Numerous studies have described the technique and clinical results of diagnostic blocks for chronic LBP (Table 4). Saal describes as the gold standard of diagnostic FJB the highly controlled (CT, MRI) FJB at the median nerve branch (MBB) [35]. Dreyfuss concludes from his meta analysis that FJB via MBB or LA-FJI has the same specificity [34], [48]. Reproducibility of the test is not high: the specificity is only 65% [72], [73]. However, the specificity of diagnostic MBB is also not high, with false-positive rates ranging from 25% to 38% [71], [74], [75]. Standard blockade injections of the medial branches seem to anesthetize the joint and also the muscles, ligaments and periosteum they innervate [74].

Despite these known neurophysiologic limitations, the known problems with validity and specificity, the FJI are commonly used for the diagnosis of suspected pathology in the FJ. But due to the discussed reasons, the single local FJB via LA as diagnostic tool for FJS has to be abandoned. Therefore, the assessment of the severity of the clinical relevant degenerative FJS and of the success of the therapy lies in the optimization of the specificity of the diagnostic tool. Leclaire et al. [76] approximately indicated in their discussion that diagnosing FJ mediated pain is more effectively done via comparative anesthetic (and saline placebo) blocks. To obtain a safe result, 3 blocks would have to be performed: one with LA and 2 with NaCl (placebo) or LA in a blinded setting [77]. The current standard of diagnosing FJ-mediated pain via comparative local anesthetic blocks with placebo-controls is exacting. And although our results show, that interpretation of these testing has its limits and that the results are not valid, it should be a standard that we must uphold for the sake of our patients because it's the only standard we have till now. There is no completely reliable gold standard with which to compare a diagnostic test (or injection) when the absence of pain is the end point [35]. A true comparison is not possible. The test results have to be interpretated in the context of all clinical and radiological findings and the somatic and psychological patient history. They are not able to give diagnosis of the FJ being a major pain generator on their own.

### Conclusions

With regard to test validity criteria, a single intraarticular facet block with local anesthetics is not useful to prove a FJS and has to be abandoned from preoperative testing and indication finding. Although several studies have been performed in the last decades, evaluation of FJI remains difficult due to lack of reliable clinical and radiological predictors. Comparative FJ blocks with local anesthetics and placebo-controls give no proper diagnosis on FJ being main pain generator. But they they are the only standard we have till now.

## References

[1] Goldthwait JE (1911). The lumbosacral articulation: an explanation of many causes of lumbago, sciatica, and paraplegia.. Boston Med Surg J.

[2] Ghormley RK (1933). Low-back pain with special reference to the articular facets, with presentation of an operative procedure.. JAMA.

[3] Hirsch C, Ingelmark B, Miller M (1963). The anatomical basis of low back pain.. Acta Orthop Scand.

[4] Mooney V, Robertson J (1976). The facet syndrome.. Clin Orthop.

[5] Jackson RP (1992). The facet syndrome. Myth or reality?. Clin Orthop.

[6] Carrera GF, Williams AL (1984). Current concepts in evaluation of the lumbar facet joints.. Crit Rev Diagn Imaging.

[7] Lewinnek GE, Warfield CA (1986). Facet joint degeneration as a cause of low back pain.. Clin Orthop.

[8] Helbig T, Lee CK (1988). The lumbar facet syndrome.. Spine.

[9] El-Khoury GY, Renfrew DL (1991). Percutaneous procedures for the diagnosis and treatment of lower back pain: diskography, facet-joint injection, and epidural injection.. AJR Am J Roentgenol.

[10] Adams MA, Hutton WC (1986). The effect of posture on diffusion into lumbar intervertebral discs.. J Anat.

[11] Adams MA, Hutton WC (1983). The mechanical function of the lumbar apophyseal joints.. Spine.

[12] Dunlop RB, Adams MA, Hutton WC (1984). Disc space narrowing and the lumbar facet joints.. J Bone Joint Surg Br.

[13] Lorenz M, Patwardhan A, Vanderby R (1983). Load-bearing characteristics of lumbar facets in normal and surgically altered spinal segments.. Spine.

[14] Yang KH, King AI (1984). Mechanism of facet load transmission as a hypothesis for low-back pain.. Spine.

[15] Gotfried Y, Bradford DS, Oegema TR (1986). Facet joint changes after chemonucleolysis-induced disc space narrowing.. Spine.

[16] North RB, Kidd DH, Campbell JN, Long DM (1991). Dorsal root ganglionectomy for failed back surgery syndrome: a five year followup study.. J Neurosurg.

[17] Ashton IK, Ashton BA, Gibson SJ, Polak JM, Jaffray DC (1992). Morphological basis for back pain: the demonstration of nerve fibers and neuropeptides in the lumbar facet joint capsule but not in ligamentum flavum.. J Orthop Res.

[18] Gronblad M, Korkala O, Konttinen YT, Nederstrom A, Hukkanen M (1991). Silver impregnation and immunohistochemical study of nerves in lumbar facet joint plical tissue.. Spine.

[19] Konttinen YT, Gronblad M, Korkala O, Tolvanen E, Polak JM (1990). Immunohistochemical demonstration of subclasses of inflammatory cells and active, collagen-producing fibroblasts in the synovial plicae of lumbar facet joints.. Spine.

[20] McLain RF, Pickar JG (1998). Mechanoreceptor endings in human thoracic and lumbar facet joints.. Spine.

[21] Giles LG, Harvey AR (1987). Immunohistochemical demonstration of nociceptors in the capsule and synovial folds of human zygapophyseal joints.. Br J Rheumatol.

[22] Bogduk N, Twomey LT (1991). Clinical anatomy of the lumbar spine. 2nd ed.

[23] Fukui S, Ohseto K, Shiotani M, Ohno K, Karasawa H (1997). Distribution of referred pain from the lumbar zygapophyseal joints and dorsal rami.. Clin J Pain.

[24] Maldjian C, Mesgarzadeh M, Tehranzadeh J (1998). Diagnostic and therapeutic features of facet and sacroiliac joint injection. Anatomy, pathophysiology, and technique.. Radiol Clin North Am.

[25] Marks RC, Houston T, Thulbourne T (1992). Facet joint injection and facet nerve block: a randomised comparison in 86 patients with chronic low back pain.. Pain.

[26] McCall IW, Park WM, O'Brien JP (1979). Induced pain referral from posterior lumbar elements in normal subjects.. Spine.

[27] Grogan J, Nowicki BH, Schmidt TA, Haughton VM (1997). Lumbar facet joint tropism does not accelerate degeneration of the facet joints.. AJNR Am J Neuroradiol.

[28] Schleifer J, Kiefer M, Hagen T (1995). Lumbar facet syndrome. Recommendation for staging before and after intra-articular injection treatment.. Radiologe.

[29] Schwarzer AC, Derby R, Aprill CN, Fortin J, Kine G (1994). Pain from the lumbar zygapophysial joints: a test of two models.. J Spinal Disord.

[30] Boden SD, Wiesel SW (1996). Lumbar Spine Imaging: Role in Clinical Decision Making.. J Am Acad Orthop Surg.

[31] Boden SD (1996). The use of radiographic imaging studies in the evaluation of patients who have degenerative disorders of the lumbar spine.. J Bone Joint Surg Am.

[32] Boden SD, Swanson AL (1998). An assessment of the early management of spine problems and appropriateness of diagnostic imaging utilization.. Phys Med Rehabil Clin N Am.

[33] Jensen MC, Brant-Zawadzki MN, Obuchowski N, Modic MT, Malkasian D (1994). Magnetic resonance imaging of the lumbar spine in people without back pain.. N Engl J Med.

[34] Dreyfuss PH, Dreyer SJ, Herring SA (1995). Lumbar zygapophysial (facet) joint injections.. Spine.

[35] Saal JS (2002). General principles of diagnostic testing as related to painful lumbar spine disorders: a critical appraisal of current diagnostic techniques.. Spine.

[36] Pfirrmann CW, Hodler J, Boos N (1999). Diagnostische Abklärung beim lumbalen Rückenschmerz.. Praxis.

[37] Schwarzer AC, Wang SC, O'Driscoll D, Harrington T, Bogduk N (1995). The ability of computed tomography to identify a painful zygapophysial joint in patients with chronic low back pain.. Spine.

[38] Jackson RP, Jacobs RR, Montesano PX (1988). 1988 Volvo award in clinical sciences. Facet joint injection in low-back pain. A prospective statistical study.. Spine.

[39] Roy DF, Fleury J, Fontaine SB, Dussault RG (1988). Clinical evaluation of cervical facet joint infiltration.. Can Assoc Radiol J.

[40] Revel ME, Listrat VM, Chevalier XJ, Dougados M, N'guyen MP (1992). Facet joint block for low back pain: identifying predictors of a good response.. Arch Phys Med Rehabil.

[41] Schwarzer AC, Aprill CN, Derby R, Fortin J, Kine G (1994). Clinical features of patients with pain stemming from the lumbar zygapophysial joints. Is the lumbar facet syndrome a clinical entity?. Spine.

[42] Carette S, Marcoux S, Truchon R, Grondin C, Gagnon J (1991). A controlled trial of corticosteroid injections into facet joints for chronic low back pain.. N Engl J Med.

[43] Schwarzer AC, Wang SC, Bogduk N, McNaught PJ, Laurent R (1995). Prevalence and clinical features of lumbar zygapophysial joint pain: a study in an Australian population with chronic low back pain.. Ann Rheum Dis.

[44] Jerosch J, Tappiser R, Assheuer J (1998). MRI-controlled facet block–technique and initial results.. Biomed Tech (Berl).

[45] Cook NJ, Hanrahan P, Song S (1999). Paraspinal abscess following facet joint injection.. Clin Rheumatol.

[46] Zennaro H, Dousset V, Viaud B, Allard m, Dehais J (1998). Periganglionic foraminal steroid injections performed under CT control.. Am J Neuroradiol.

[47] Esses SI, Moro JK (1993). The value of facet joint blocks in patient selection for lumbar fusion.. Spine.

[48] Marks R (1989). Distribution of pain provoked from lumbar facet joints and related structures during diagnostic spinal infiltration.. Pain.

[49] Jerosch J (1994). Das Facettensyndrom.

[50] Grönemeyer D, Seibel R (1989). Interventionelle Computertomographie.

[51] Moran R, O'Connell D, Walsh MG (1988). The diagnostic value of facet joint injections.. Spine.

[52] White AH (1983). Injection techniques for the diagnosis and treatment of low back pain.. Orthop Clin North Am.

[53] Purcell-Jones G, Pither CE, Justins DM (1989). Paravertebral somatic nerve block: a clinical, radiographic, and computed tomographic study in chronic pain patients.. Anesth Analg.

[54] Alcock E, Regaard A, Browne J (2003). Facet joint injection: a rare form cause of epidural abscess formation.. Pain.

[55] Magee M, Kannangara S, Dennien B, Lonergan R, Emmett L (2000). Paraspinal abscess complicating facet joint injection.. Clin Nucl Med.

[56] Orpen NM, Birch NC (2003). Delayed presentation of septic arthritis of a lumbar facet joint after diagnostic facet joint injection.. J Spinal Disord Tech.

[57] Marks R, Semple AJ (1992). Chemical meningism after lumbar facet joint block.. Anaesthesia.

[58] Marks R, Semple AJ (1988). Spinal anaesthesia after facet joint injection.. Anaesthesia.

[59] Goldstone JC, Pennant JH (1987). Spinal anaesthesia following facet joint injection. A report of two cases.. Anaesthesia.

[60] Okazaki K, Sasaki K, Matsuda S, Yuge I, Omiya K (2000). Pyogenic arthritis of a lumbar facet joint.. Am J Orthop.

[61] Fairbank JC, Park WM, McCall IW, O'Brien JP (1981). Apophyseal injection of local anesthetic as a diagnostic aid in primary low-back pain syndromes.. Spine.

[62] Schuirmann DJ (1987). A comparison of the two one-sided tests procedure and the power approach for assessing the equivalence of average bioavailability.. J Pharmacokinet Biopharm.

[63] Agorastides ID, Kumar N (2001). The oblique needle technique in lumbar facet joint injection.. Eur J Radiol.

[64] Glover JR (1977). Arthrography of the joints of the lumbar vertebral arches.. Orthop Clin North Am.

[65] Dory MA (1981). Arthrography of the lumbar facet joints.. Radiology.

[66] Eisenstein SM, Parry CR (1987). The lumbar facet arthrosis syndrome. Clinical presentation and articular surface changes.. J Bone Joint Surg Br.

[67] Destouet JM, Gilula LA, Murphy WA, Monsees B (1982). Lumbar facet joint injection: indication, technique, clinical correlation, and preliminary results.. Radiology.

[68] Hildebrant J (2000). Relevance ofinvasive techniques in treating and diagnosing chronic low back pain: an anesthesiologic perspective.. Sem Spine Surg.

[69] Lilius G, Laasonen EM, Myllynen P, Harilainen A, Gronlund G (1989). Lumbar facet joint syndrome. A randomised clinical trial.. J Bone Joint Surg Br.

[70] Lynch MC, Taylor JF (1986). Facet joint injection for low back pain. A clinical study.. J Bone Joint Surg Br.

[71] Schwarzer AC, Aprill CN, Derby R, Fortin J, Kine G (1994). The false-positive rate of uncontrolled diagnostic blocks of the lumbar zygapophysial joints.. Pain.

[72] Meier G, Büttner J (2001). Regionalanästhesie. Kompendium der peripheren Blockaden.

[73] Carragee EJ, Hannibal M (2004). Diagnostic evaluation of low back pain.. Orthop Clin North Am.

[74] Murtagh R (2000). The art and science of nerve root and facet blocks.. Neuroimaging Clin N Am.

[75] Van Kleef M, Barendse GA, Kessels A, Voets HM, Weber WE (1999). Randomized trial of radio frequency lumbar facet denervation for chronlc low back pain.. Spine.

[76] Leclaire R, Fortin L, Lambert R, Bergeron YM, Rossignol M (2001). Radiofrequency facet joint denervation in the treatment of low back pain.. Spine.

[77] Hildebrandt J (2001). Relevance of nerve blocks in treating and diagnosing low back pain–is the quality decisive?. Schmerz.

